# Structural basis for host recognition and superinfection exclusion by bacteriophage T5

**DOI:** 10.1073/pnas.2211672119

**Published:** 2022-10-10

**Authors:** Bert van den Berg, Augustinas Silale, Arnaud Baslé, Astrid F. Brandner, Sophie L. Mader, Syma Khalid

**Affiliations:** ^a^Biosciences Institute, The Medical School, Newcastle University, Newcastle upon Tyne NE2 4HH, United Kingdom;; ^b^Department of Biochemistry, University of Oxford, Oxford OX1 3QU, United Kingdom

**Keywords:** bacteriophage T5, TonB-dependent transporter, lipoprotein, superinfection exclusion, FhuA

## Abstract

A key but poorly understood stage of the bacteriophage life cycle is the binding of phage receptor-binding proteins (RBPs) to receptors on the host cell surface, leading to injection of the phage genome and, for lytic phages, host cell lysis. To prevent secondary infection by the same or a closely related phage and nonproductive phage adsorption to lysed cell fragments, superinfection exclusion (SE) proteins can prevent the binding of RBPs via modulation of the host receptor structure in ways that are also unclear. Here, we present the cryogenic electron microscopy (cryo-EM) structure of the phage T5 outer membrane (OM) receptor FhuA in complex with the T5 RBP pb5, and the crystal structure of FhuA complexed to the OM SE lipoprotein Llp. Pb5 inserts four loops deeply into the extracellular lumen of FhuA and contacts the plug but does not cause any conformational changes in the receptor, supporting the view that DNA translocation does not occur through the lumen of OM channels. The FhuA–Llp structure reveals that Llp is periplasmic and binds to a nonnative conformation of the plug of FhuA, causing the inward folding of two extracellular loops via “reverse” allostery. The inward-folded loops of FhuA overlap with the pb5 binding site, explaining how Llp binding to FhuA abolishes further infection of *Escherichia coli* by phage T5 and suggesting a mechanism for SE via the jamming of TonB-dependent transporters by small phage lipoproteins.

## Significance

Bacteriophages, viruses that prey on bacteria, are the most abundant organisms on Earth and play a crucial role in shaping microbial ecosystems. A key event, still poorly understood at the molecular level, in the bacteriophage life cycle is the interaction of phage receptor-binding proteins with receptors on the bacterial cell surface, leading ultimately to phage propagation. Another important and understudied process in phage biology is superinfection exclusion (SE), which prevents secondary infections by the same or similar viruses. Here, we visualize, for the model phage T5, how host recognition and SE can be achieved via the interaction of different phage proteins with the same receptor in the *Escherichia coli* outer membrane.

The increasing threat posed by multidrug-resistant bacteria, coupled with the lack of novel antibiotics, has led to a resurgent interest in the potential use of phage therapy to treat bacterial infections (1, 2), including phage steering (3). Notwithstanding the enormous variety in phage structure and function, a defining moment during the infectious cycle of any phage is the high-affinity binding to protein and/or nonprotein receptors on the host cell surface by phage receptor-binding proteins (RBPs) (4). This causes adsorption of the phage on the cell surface and leads to the injection of the phage genome into the bacterial cell via a sequence of events that is still poorly understood. For lytic phages, genome injection leads to the assembly of progenitor phage in the host cytoplasm and cell lysis. To prevent the nonproductive adsorption of phage particles to already infected cells and postlysis cell fragments, many phages express superinfection exclusion (SE) proteins early during infection (5). One way to achieve SE is by inactivating the target receptor for RBPs. Similar to host protein receptor binding by RBPs, the mechanism by which SE proteins inactivate those receptors has not yet been visualized for any phage.

**Fig. 1.**
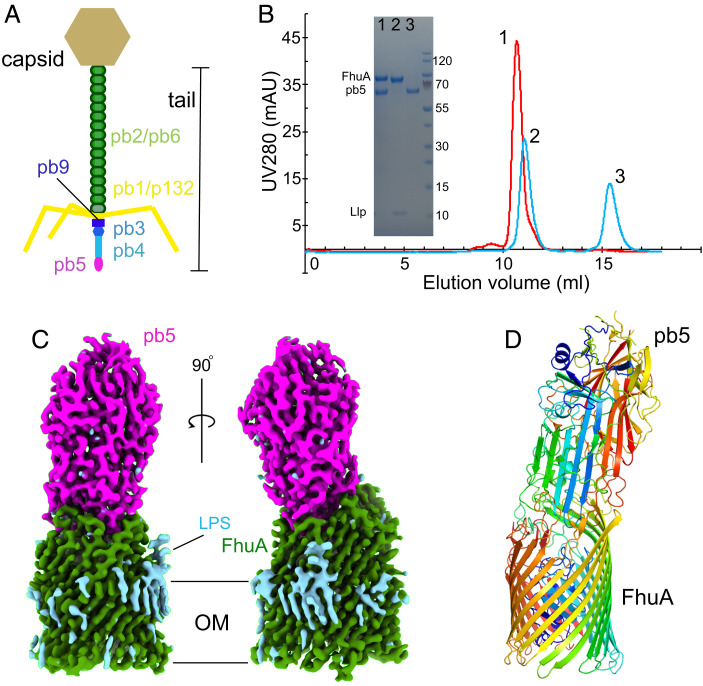
Cryo-EM structure of the FhuA–pb5 complex. (*A*) Schematic representation of bacteriophage T5. (*B*) Analytical SEC profiles for samples containing FhuA plus pb5 (red) and FhuA–Llp plus pb5 (cyan). One nanomole of each protein was used. Peaks are numbered and were analyzed via sodium dodecyl sulfate–polyacrylamide gel electrophoresis (*Inset*). Band identities (*Left*) and the size of molecular weight markers (*Right*) are shown. Curves shown are representative for three separate experiments. (*C*) Cryo-EM maps of FhuA–pb5 shown within the OM plane. FhuA density is colored green and pb5 density is in magenta. LPS or detergent density is in cyan. (*D*) Cartoon model of FhuA–pb5 colored in rainbow representations (N termini are in blue). mAU, milli-absorbance unit.

The lytic bacteriophage T5 is one of the model T coliphages that have been studied in great detail and which are the basis of many fundamental discoveries in molecular biology (6–8). Phage T5 is a caudal (tailed) virus within the family Demerecviridae and was sequenced in 2005 (9). Of its 162 predicted open reading frames (9), more than half lack similarity to known genes, and many T5 proteins are still uncharacterized, a common theme in phage biology. The overall morphology of T5 has been visualized via cryogenic electron microscopy (cryo-EM) (10). As a Siphovirus, T5 has a long and flexible noncontractile tail (∼160 nm long) composed of the tail tube protein pb6 (11) and containing the tape measure protein pb2 that most likely perforates the *Escherichia coli* cell envelope (Fig. 1*A*) (12). The distal tail tip connects to a baseplate that anchors the three lateral tail fibers (13) composed of pb1 that bind lipopolysaccharide (LPS) O-antigen reversibly (14). The baseplate is composed of the distal tail tip protein pb9 and the baseplate hub protein pb3 that leads to the central straight fiber pb4 (Fig. 1*A*). Last, the RBP pb5 (*oad* gene; Uniprot P23907) mediates irreversible T5 adsorption to *E. coli* cells (15) and is likely located at the distal end of pb4 (Fig. 1*A*). With exception of the monomeric RBP pb5, all tail proteins likely form oligomers within the intact phage (13).

The receptor for phage T5 was identified as the outer membrane (OM) TonB-dependent transporter (TBDT) FhuA almost 50 y ago (16, 17). Purified FhuA and pb5 form a highly stable complex (18, 19) that was characterized at low resolution by small-angle neutron scattering and negative-stain electron microscopy (20). Taking into account the low resolution, no major conformational changes were observed upon complex formation, contrasting with multicopy RBPs that bind to surface polysaccharides with low affinities (21). Addition of purified pb5 to *E. coli* cells blocks subsequent T5 infection and affects other processes that depend on functional FhuA, such as ferrichrome import (19). Similar phenotypes are observed upon expression of the small phage lipoprotein Llp (Uniprot Q38162), which is adjacent and downstream to pb5 on the T5 genome, establishing it as the phage T5 SE protein (22–24). Llp, also termed lytic conversion protein (22), has low sequence similarity to the small Cor lipoproteins that function as SE proteins in T1 and related coliphages (25). An in vitro study placed Llp on the outside of the cell (24), but in vivo work suggested Llp is periplasmic (23). A model was proposed in which Llp, by binding to FhuA, would cause allosteric conformational changes in the pb5 binding site on the extracellular surface, thereby preventing T5 binding (23).

To elucidate the mechanism of SE via potential TBDT structure modulation, we report here the cryo-EM structure of the FhuA–pb5 complex and the X-ray crystal structure of the FhuA–Llp complex. The FhuA–Pb5 structure shows that pb5 is an elongated molecule with one end inserted into the extracellular lumen of the FhuA barrel and with its long axis approximately perpendicular to the OM plane. All extracellular FhuA loops except EL1 toEL3 contact pb5, providing an qualitative explanation for the high stability of the interaction. Free FhuA and FhuA within the complex are virtually identical. The domain of pb5 that interacts with pb4 is poorly ordered, providing a possible mechanism for transmitting a conformational change from pb5 to the rest of the tail. The FhuA–Llp structure shows that Llp is, indeed, bound to the periplasmic face of FhuA, making extensive interactions with the FhuA plug. Strikingly, the conformation of the plug within the complex is nonnative, suggesting that Llp has bound to an FhuA intermediate state during TonB-dependent transport. On the extracellular side, FhuA loops EL7 and EL8 have undergone large conformational changes to fold inward and completely block access to the plug domain. EL7 and EL8 would clash with pb5, providing an explanation for small lipoprotein-mediated SE via modulation of receptor structure.

## Results

To explore the pb5/FhuA/Llp interactions in vitro, individual components were expressed in *E. coli* and purified via immobilized metal affinity chromatography (IMAC) and size exclusion chromatography (SEC). While pb5 is a soluble protein and does not stably associate with detergent micelles (unlike Llp and FhuA), the addition of detergent to *E. coli* lysates improved pb5 yield and behavior on SEC, and pb5 was therefore purified in the presence of detergent. For FhuA–Llp, we observed that complex formation via addition of purified Llp to FhuA is very slow (*SI Appendix*, Fig. S1), suggesting that the FhuA conformation to which Llp binds is poorly accessible or sparsely populated in vitro. To obtain the FhuA–Llp complex, we co-overexpressed FhuA and Llp on different plasmids in *E. coli* and purified the in vivo–assembled complex via IMAC and SEC. Depending on the particular preparation, we could obtain a roughly equimolar complex that is very stable during gel filtration (*SI Appendix*, Fig. S1).

We next analyzed the interaction of the proteins via SEC. Mixing of equimolar amounts of pb5 and FhuA followed by short incubation (5 min) results in one peak on SEC containing both components (Fig. 1*B*) and no trace of free pb5, indicating formation of a very stable FhuA–pb5 complex. By contrast, mixing equimolar amounts of pb5 and FhuA–Llp yields two well-separated peaks on SEC, with no pb5 coeluting with FhuA–Llp. It should be noted that pb5 elutes much later than expected on SEC, and the FhuA–pb5 complex runs only slightly faster than FhuA alone (Fig. 1*B*). These data show that the prevention of pb5 binding to the phage T5 receptor FhuA by the phage lipoprotein Llp can be reconstituted in vitro with purified components and that no other factors are required.

**Fig. 2.**
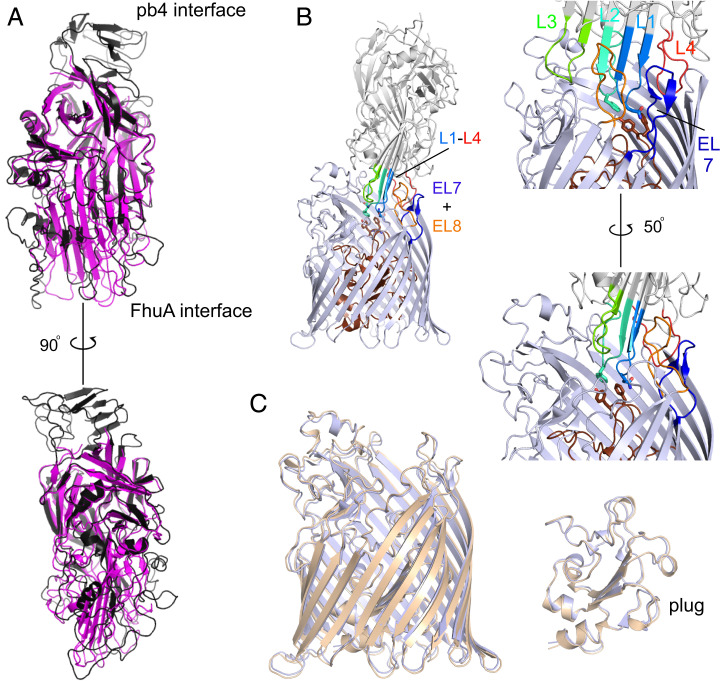
Structural analysis of the FhuA–pb5 interaction. (*A*) Superpositions of AF2-predicted pb5 (black) and pb5 bound to FhuA (magenta). Note the absence of the putative pb4-interacting domain of pb5 within the complex. (*B*) Cartoon viewed from the OM plane, highlighting the interacting loops from pb5 (L1 to L4) and FhuA (EL7 and EL8). (*Right*) Two close-up views. Residues Gln115/Phe-170 of pb5 and Phe115/Tyr116 of FhuA are shown as stick models. The FhuA plug is colored brown. (*C*) Superposition of free FhuA (crystal structure; PDB ID 1BY3, shown in light brown) and FhuA within the FhuA–pb5 complex (light blue).

Since pb5 bound to FhuA is more stable at high concentrations (>0.5 mg/mL) than pb5 in isolation, the FhuA–pb5 complex was obtained in milligram amounts by mixing prepurified FhuA with *E. coli* cell lysates expressing pb5, followed by IMAC and gel filtration in the presence of detergent (Fig. 1*B*). Crystallization trials yielded crystals diffracting anisotropically and only to modest resolutions (∼4 Å), and the phase problem could not be solved by molecular replacement (MR) with FhuA (Protein Data Bank identifier [PDB ID] 1BY3) (26) and AF2-predicted pb5 as search models (27, 28). We succeeded in solving the FhuA–pb5 structure via cryo-EM, using complex purified in decylmaltopyranoside at ∼3.1 Å resolution (*SI Appendix*, Table S1 and Fig. S2) (29, 30). The ∼150 kDa complex is ∼150 Å high and has a largest width of ∼65 Å at the base of the FhuA barrel (Fig. 1*C*). The LPS molecule that is present in FhuA X-ray crystal structures (31) is clearly visible in the cryo-EM map. Consistent with the AF2 prediction of isolated pb5, FhuA-bound pb5 has an oblong shape with a large central β-sheet (Fig. 1*D*). Like many phage proteins, pb5 is not similar to any other protein; a distance matrix alignment analysis (32) identifies PDB ID 2GSY (polyprotein) as the closest structural homolog, with a Z-score of 5.3 but with only 99 aligned residues (α carbon root-mean-square deviation [Cα RMSD], 3.5 Å; *SI Appendix*, Fig. S3). The central region of pb5 is virtually identical to that in the AF2 prediction, while the part that interacts with FhuA shows large differences (overall RMSD, 1.7 Å for 529 of 640 Cα atoms; Fig. 2*A* and *SI Appendix*, Fig. S4). Interestingly, the region at the other end of pb5 that most likely interacts with pb4 is poorly ordered but present in the cryo-EM density and only visible at low contours (*SI Appendix*, Fig. S4). The predicted structure of this region shows pseudo threefold symmetry, suggesting that pb4 may be trimeric. Overall, 529 of 640 pb5 residues could be modeled.

Pb5 inserts four loops (numbered L1 to L4 from the N terminus) into the extracellular lumen of FhuA (Fig. 2*B* and Movie S1). The involvement of L4 (residues 570 to 590) adjusts the previous assignment of the N-terminal half of pb5 as the FhuA-interacting domain (33). The conformations of the binding loops, as well as those of the other loops of pb5 that interact with FhuA, are predicted with low confidence by AF2, so it is unclear whether their very different conformations within the complex are caused by the FhuA interaction (Fig. 2*A* and *SI Appendix*, Fig. S4). Pb5 residues Gln115 in the tip of L1 and Phe170 in the tip of L2 contact Phe115 and Tyr116 in the FhuA plug (residue numbering for the mature part of FhuA), but it is clear that the insertion of pb5 does not affect the position and conformation of the plug (Fig. 2*C*). In fact, the entire FhuA structure remains remarkably similar upon pb5 binding, as judged from a comparison with the FhuA crystal structure (Cα RMSD, 0.8 Å with 1BY3; Fig. 2*C*). With the exception of EL1-EL3, all FhuA loops contact pb5, resulting in a large interface area of ∼2140 Å^2^ as analyzed via PISA (34). There are 27 intermolecular hydrogen bonds, with most of them between EL4-L2 (8), EL5-L2 (8), and EL8-L1-3 (7) (*SI Appendix*, Fig. S5*A*). Our structure supports the data from a study on the effect of systematic FhuA loop deletions on T5 infection, where deletion of any individual loop, with the exception of EL8, had only a modest effect on T5 sensitivity (35). Pb5 completely fills the extracellular lumen of FhuA and occludes the ferrichrome binding site (*SI Appendix*, Fig. S6), explaining why addition of purified pb5 to *E. coli* inhibits growth under iron-starved conditions (19). Interestingly, pb5 binding does not cause conformational changes in the TonB box, and the structure of the plug in FhuA–pb5 is identical to that in apo FhuA, despite the fact that ferrichrome and pb5 both contact Phe115 and Tyr116 of the plug (*SI Appendix*, Fig. S6). Thus, phage T5 is not recognized as a ligand by FhuA.

**Fig. 3.**
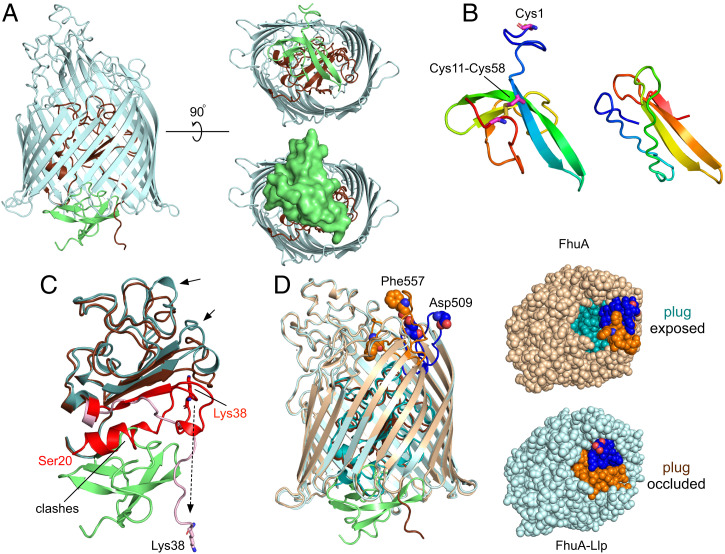
Llp binding to FhuA generates allosteric conformational changes in extracellular loops. (*A*) Cartoon viewed from the OM plane showing Llp bound to the periplasmic face of FhuA (light blue; plug, brown). Llp is colored lime green. (*Right*) Views from the periplasmic space, with a surface model of Llp shown at the *Bottom*. (*B*) Comparison of Llp bound to FhuA (*Left*) and AF2-predicted Llp (*Right*). Views were generated from a superposition. The predicted Llp model does not have the Cys11-Cys58 disulphide bond constraining the termini. (*C*) Llp binds to a nonnative state of the FhuA plug. The plug of free FhuA (PDB ID 1B3Y) is teal, with residues Ser20-Glu57 colored red. The plug of Llp-bound FhuA is in brown, with residues Lys38-Glu57 in pink. Residue Lys38 is shown for both structures as sticks. “Clashes” indicates the region of the free FhuA plug that would overlap with Llp. Arrows show movements of plug segments on the extracellular side of the plug. View direction is roughly as in the *Left* panel of *A*, with the periplasmic space at the bottom. (*D*) Extracellular changes in FhuA caused by Llp binding. Superposition of free FhuA (light brown) and FhuA–Llp (light blue), with their plug domains colored in teal and brown, respectively. FhuA loops EL7 (Glu501-Ser516) and EL8 (Thr546-Glu564) are colored blue and orange, respectively, with residues Asp509 (EL7) and Phe557 (EL8) shown as space-filling models and labeled for free FhuA. (*Right*) Space-filling models from the outside of the cell, demonstrating the occlusion of the plug by EL7 and EL8 movement resulting from Llp binding.

Having characterized the FhuA–pb5 interaction at high resolution, we next focused on solving the structure of the FhuA–Llp complex. Depending on the particular preparation, we could obtain a roughly equimolar complex that is stable during gel filtration. Due to the relatively small size of FhuA–Llp (∼90 kDa), we utilized X-ray crystallography for this part of the project. Extensive screening yielded one crystal form that contained both components (*SI Appendix*, Fig. S1) and had useful, anisotropic diffraction to ∼3.4 Å resolution. MR with FhuA resulted in maps with unaccounted density on the periplasmic face of FhuA but of insufficient quality for model building. Adding an AF2-predicted Llp model to the MR search gave a solution that allowed building and refinement of the complete FhuA–Llp complex (Fig. 3*A* and *SI Appendix*, Table S2) (36). The structure shows that the AF2 prediction of free Llp, while providing valuable phasing information, is inaccurate overall (Cα RMSD, 2.8 Å for 28 aligned atoms out of 61; Fig. 3*B*). Interestingly, the AF2-predicted complex places Llp on the extracellular side. However, the position of Llp in the prediction is impossible to reconcile with a lipid anchor on Cys1, even when assuming that Llp is flipped across the OM to the cell surface (*SI Appendix*, Fig. S7). To investigate the unlikely scenario that the lipid anchor of Llp is cleaved off after OM flipping, generating soluble Llp that could bind to FhuA extracellularly, we performed unbiased molecular dynamics (MD) simulations of the AF2 complex (*SI Appendix*, Fig. S8). The data clearly show that, compared with simulations of the experimental FhuA–Llp structure, Llp is relatively unstable in the AF-predicted binding site. This might provide an explanation for the claim by Pedruzzi et al. (24) that a ratio of soluble Llp to FhuA of 10^6^ was required to abolish phage T5 binding in vitro. Such ratios are unlikely to be physiological, and it is clear that, in vivo, Llp is lipidated and located in the periplasmic space.

Llp is bound to the periplasmic face of FhuA and makes extensive interactions with both the plug and the barrel. The total interface area is 1,640 Å^2^, with 14 hydrogen bonds and four salt bridges (*SI Appendix*, Fig. S5B). Many of the interactions occur between Llp and the visible N-terminal ∼20 residues of the plug, comprising Lys38-Glu57. Interestingly, those plug residues have a very different conformation in free FhuA. In addition, density up to Ser20 is visible in free FhuA, which includes the N-terminal switch helix. Pairwise backbone differences between residues visible in both structures are as much as 26 Å for Lys38 (Fig. 3*C*). Strikingly, several Llp residues (Ile39-Trp46) occupy space where the N-terminal switch helix of free FhuA would be, indicating that Llp has bound to a nonnative conformation of FhuA (Fig. 3*C* and *SI Appendix*, Fig. S9). Structural changes in the rest of the plug are less extensive but nevertheless still include backbone shifts of 3 to 4 Å downward, toward the periplasmic space.

**Fig. 4.**
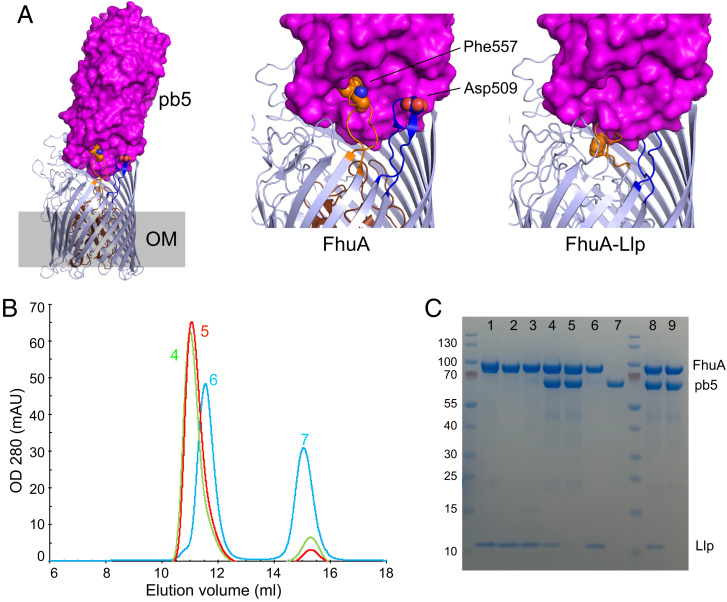
Llp-induced movements of FhuA extracellular loops abolish pb5 binding. (*A*) Side view of FhuA with the pb5 surface superimposed. Close ups of the EL7/EL8 interface with pb5 for free FhuA (*Center*) and FhuA–Llp (*Right*). (*B*) Analytical SEC profiles for samples containing FhuA ΔEL7 plus pb5 (red), FhuA ΔEL7–Llp plus pb5 (green), and wild-type FhuA–Llp plus pb5 (cyan). Samples contained 1.5 nmol of each protein. Curves shown are representative for three separate experiments. (*C*) Sodium dodecyl sulfate–polyacrylamide gel electrophoresis analysis of protein fractions, as follows: lane 1, stoichiometric amounts of FhuA and Llp, purified separately; lane 2, wild-type FhuA–Llp (coexpressed); lane 3, FhuA ΔEL7–Llp (coexpressed); lane 4, FhuA ΔEL7–Llp (preparation 1) plus pb5; lane 5, FhuA ΔEL7 (preparation 1) plus pb5; lane 6 and 7, wild-type FhuA–Llp plus pb5; lane 8, FhuA ΔEL7–Llp (preparation 2) plus pb5; and lane 9, FhuA ΔEL7 (preparation 2) plus pb5. Band identities are shown on the *Right* and the size (kDa) of molecular weight markers on the *Left*. mAU, milli-absorbance unit.

On the extracellular side, the conformational changes in FhuA–Llp relative to free FhuA are dramatic but confined to just two loops, EL7 and EL8 (Fig. 3*D*). Both loops fold inward to completely occlude the plug domain in Llp-bound FhuA. Backbone shifts for residues located at the loop tips (Asp509 in EL7 and Phe557 in EL8) are ∼15 Å (Fig. 3*D* and Movie S2). Interestingly, the deletion of EL8 has the biggest effect on phage T5 sensitivity of all FhuA loops (35), potentially explaining why this particular loop, together with EL7, undergoes a conformational change on Llp binding. To investigate the possibility that the conformations of EL7 and EL8 are induced by the crystallization process, we performed unbiased MD simulations on the FhuA–Llp system. Three independent simulations show that the loop conformations are stable on the timescales of the simulations (2 μs; *SI Appendix*, Fig. S10), supporting the notion they have been induced by Llp binding to FhuA and not by the crystal lattice. Llp is mobile in the simulations but remains bound to FhuA, suggesting that the interactions of Llp with the plug dominate those with the FhuA barrel. Importantly, the effect of the EL7 and EL8 movements is that they will prevent the high-affinity binding of pb5 to FhuA because of extensive clashes with the FhuA binding loops of pb5 (Fig. 4*A*), explaining our in vitro data with purified components and in vivo data from the literature (Fig. 1*B*) (19, 22–24). Moreover, the Llp-induced loop positions will also prevent albomycin binding (*SI Appendix*, Fig. S6*E*) (37), explaining why cells coexpressing FhuA and Llp become resistant toward this antibiotic (23). Llp binding to FhuA also generates resistance of *E. coli* toward phage Φ80 and colicin M (23), suggesting that FhuA loops EL7 and EL8 are also involved in their binding.

To obtain additional evidence for the importance of the Llp-induced movements of EL7 and EL8 on pb5 binding, we generated the FhuA loop deletion variants ΔEL7, ΔEL8, and ΔEL7/ΔEL8. The variants with EL8 deletions expressed only to very low levels and could not be purified, but FhuA ΔEL7 expression was reasonable. The ΔEL7 mutant still formed an approximately stoichiometric complex with Llp when coexpressed (Fig. 4*B*, lane 3). In addition, FhuA ΔEL7 still bound pb5 efficiently (Fig. 4 *B*, lanes 5 and 9 and *C*). Strikingly, and in contrast to wild-type FhuA–Llp, two independently purified batches of FhuA ΔEL7–Llp still bound pb5 (Fig. 4*B*, lanes 4 and 8). These data suggest that the removal of EL7 prevents the Llp-induced inward movement of EL8, allowing pb5 to bind to the FhuA mutant in the presence of Llp. Moreover, these data support our notion that Llp is periplasmic and not extracellular, because pb5 binding to FhuA ΔEL7–Llp would otherwise be unlikely.

## Discussion

**Fig. 5.**
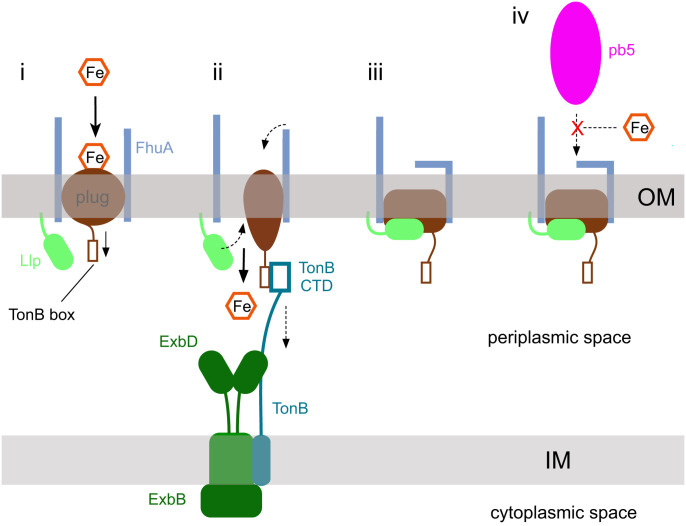
Schematic model for lipoprotein-mediated SE via TBDTs. (i) Substrate (in this case, iron siderophore) binding to the TBDT (FhuA; light blue) causes allosteric changes in the plug (brown) that expose the TonB box to the periplasmic space. (ii) The C-terminal domain (CTD) of TonB binds the TonB box and, likely via mechanical force generated by ExbBD, causes a conformational change or unfolding of the plug that allows substrate passage into the periplasmic space. (iii) During the transport cycle, Llp (lime green) binds to the unfolded plug, causing allosteric changes that generate conformational changes in one or more extracellular loops. The conformational changes abolish binding of phage RBPs, here represented by pb5 (iv). The SE lipoprotein likely jams the TBDT in a transport-incompetent state despite the TonB box being accessible. IM, inner membrane.

An early in vivo study (23) that assigned a periplasmic location to Llp investigated the effect of FhuA mutations on FhuA-dependent processes such as phage T5 sensitivity in the presence and absence of Llp. Unfortunately, this study, like others performed around the same time, predated the crystal structures of FhuA and assumed a wrong topology model, resulting in many mutants that were different from intended and making it challenging to rationalize the observed phenotypes. A notable exception is the I9P mutation made for the TonB box of FhuA, which is the only mutant to prevent the inactivation of FhuA by Llp (i.e., FhuA I9P cells remain fully sensitive toward T5 infection in the presence of Llp) (23). The proline substitution disrupts the β-strand structure of the TonB box and likely abolishes the interaction with TonB, as suggested by the fact that the cells become resistant to albomycin (23). Since neither Llp nor pb5 interacts with the TonB box, the FhuA I9P data support our hypothesis that Llp binds to an intermediate state of FhuA that occurs during the TonB-dependent transport cycle (Fig. 5). The TonB box in FhuA–Llp is not visible, presumably owing to flexibility, but given the exposed location of Lys38 (the first visible residue for FhuA within the complex), it seems reasonable to assume the TonB box is accessible to TonB in FhuA–Llp (*SI Appendix*, Fig. S11). We propose that Llp has jammed the plug of FhuA and that any TonB pulling does not remove the phage lipoprotein from the transporter.

Our structures show that the phage lipoprotein Llp exploits the known allostery in the plug of FhuA in the opposite direction as during ferrichrome import (i.e., from the periplasmic side to the extracellular side). How exactly Llp binding causes the conformational changes in EL7 and EL8 remains difficult to answer. The plug loops indicated by the arrows in Fig. 2*C* contact the base of EL7 and EL8, and their downward movement in FhuA–Llp creates space that may allow EL7 and EL8 to fold inward. The tips of EL7 and EL8 make many interactions with barrel and loop residues; in particular, the side chains of Phe557 and Phe558 in EL8 interact with an extensive aromatic patch, stabilizing the inward-folded conformation of EL8.

Llp has low pairwise sequence identity (∼20%, including the intramolecular disulphide bond) to members of the Cor small-lipoprotein family (25) that mediate SE in, for example, phage T1 and Φ80. These phages, like T5, use FhuA as terminal receptor, and their Cor proteins likely function in a similar way as Llp. Since the “forward” allostery in TBDTs leads to TonB box exposure in the periplasmic space regardless of the identity of transporter and substrate, it seems plausible that the lipoprotein-mediated SE we describe for FhuA is utilized by many other TBDTs. Indeed, a recent study suggests that TBDT-targeting SE lipoproteins are common in coliphages (38).

Considering phage diversity and the notion that SE seems advantageous for the phage, especially regarding prevention of nonproductive phage adsorption, it is likely that there are many ways by which SE can be achieved. As an example, a recent study showed that SE mediated by the phage T4 protein Spackle is caused by the binding of Spackle to the lysozyme domain of the T4 tail spike protein gp5, inhibiting its activity (39). While little is currently known about SE proteins, a common feature seems to be their small size, and it is conceivable that a considerable fraction of phage proteins of unknown function, many of which are small (<10 kDa), may be involved in SE. Regarding the SE mechanism we report on here, in which an OM lipoprotein modulates the structure of a protein receptor, an intriguing question is whether this also occurs in phages that have non-TBDTs as receptors, and how the cognate RBPs are blocked. Maffei et al. (38) recently characterized the host specificity of a new library of coliphages experimentally, showing that while most siphophages infecting *E. coli* use a TBDT as terminal receptor (e.g., FhuA, FepA, BtuB), a substantial number of phages target LamB, TolC, or LptD, none of which are TBDTs. The putative RBPs and cognate SE proteins of most of these phages can be identified based on their location directly downstream of the *gpJ* locus, which encodes a tail tip protein homologous to the tail J protein of phage λ. Inspection of the downstream region of *gpJ* reveals that phages that target LamB (e.g., Bas23) or LptD (e.g., Bas18 and phage RTP) likely do have SE lipoproteins (38). Since neither LamB nor LptD are known to have allostery, the question remains how those lipoproteins could block the phage receptor. *E. coli* can flip “standard” lipoproteins such as Braun's lipoprotein to the cell surface (40), so SE lipoproteins blocking, for example, LptD may be located on the extracellular side of the OM rather than being periplasmic.

A major question is how siphophage DNA is transferred into the host cell. To our knowledge, our structural data for FhuA–pb5 provide the most direct evidence to date for the view that DNA transfer does not occur via the FhuA channel, as originally proposed based on single-channel electrophysiology (41, 42). Contrasting with an earlier proposal based on cryo-ET (43), our structure shows that pb5 is bound in the center of FhuA, not at the periphery, confirming previous negative-stain electron microscopy findings (20). Combined with data placing pb5 at the very tip of the central tail fiber (Fig. 1*A*), and the loss of tail tips when detergent-purified FhuA is incubated with T5 (13), we propose that the disorder observed in the pb4-interacting part of pb5 (*SI Appendix*, Fig. S4) is caused by FhuA binding and leads to the dissociation of the tail tip. This would then result in the tape measure protein (pb2), located within the pb6 tail tube, becoming available for cell-envelope perforation and subsequent DNA injection via a mechanism that remains largely obscure.

## Materials and Methods

### Cloning, Protein Expression and Purification.

The mature part of *E. coli* FhuA (i.e., residue 34 was renumbered as 1 for compatibility with previous studies) was amplified from *E. coli* genomic DNA and cloned into a modified version of the pET9 vector via ligation-independent cloning, generating a construct with the PelB signal sequence followed by a His10 tag and tobacco etch virus (TEV) cleavage site. Site-directed mutants were generated with the Q5 mutagenesis kit (NEB). For ΔEL7, residues Ser503-Ala514 were replaced with two glycine residues. In the case of ΔEL8, the segment Met550-Glu561 was removed. The gene coding for pb5 was amplified by PCR, digested with NcoI and XhoI, and ligated into pET28-b restricted with the same enzymes, adding the sequence “LE” followed by a His6 tag to the C terminus of the protein. For Llp, a codon-optimized gene (Eurofins Genomics) for expression in *E. coli* was digested with EcoRI and XbaI and ligated into the arabinose-inducible vector pB22 restricted with the same enzymes. A C-terminal His6 tag was included in the synthetic gene.

FhuA expression was performed in the Bl21 (DE3) Δ*cyo* strain that has a clean deletion of the *cyoAB* ubiquinol oxidase genes, abolishing contamination of IMAC samples with the abundant Cyo complex and removing the need for selective removal of IM proteins via, for example, sarkosyl pre-extraction steps. Cells were grown in LB at 37 °C and 180 rpm to an optical density at 600-nm wavelength (OD_600_) of 0.2 to 0.4 (50 μg/mL kanamycin), and placed in the cold room for 20 to 30 min prior to induction with 0.2 mM isopropyl β-d-1-thiogalactopyranoside (IPTG). The cells were grown for another 18 to 20 h at 18 °C and 150 rpm. Llp was expressed either in isolation or together with FhuA in Bl21 (DE3) Δ*cyo*, as described above. Growth of cells at 37 °C gave substantially greater yields of Llp compared with growth at lower temperatures; therefore, all coexpressions of FhuA and Llp were done at 37 °C. For coexpression, Bl21 (DE3) Δ*cyo* cells were transformed with pET9 and pB22 plasmids via electroporation of freshly made electrocompetent cells and plated out on LB plates with kanamycin (50 μg/mL) and ampicillin (100 μg/mL). For liquid cultures, 35 to 40 μg/mL kanamycin was used to avoid excessive lag phases, and cells were induced with 0.2 mM IPTG and 0.1% (wt/vol) (l)-arabinose. Expression of pb5 was performed in Bl21 (DE3) at 18 °C as described above for FhuA. Final OD_600_ values were typically 2 to 3.

Cells were harvested by centrifugation for 20 min at 4,200 rpm (JS 4.2 rotor) in a Beckman J6-HC centrifuge. Cell pellets were processed either directly or frozen at −20 °C. Cells were resuspended in TSB buffer (20 mM Tris, 300 mM NaCl, pH 7.8), homogenized by douncing, and lysed in a cell disrupter (Constant Systems, 0.75 kW model) at 20 to 23,000 psi (one pass) in the presence of DNase. Typically 120 mL of buffer was used for 2 to 4 L of cells. The lysed cells were centrifuged at 42,000 rpm (45Ti rotor; Beckman Optima XE-90) for 50 min and either the supernatant (in the case of pb5) or the total membrane pellet (for FhuA and/or Llp) was collected. Membranes containing overexpressed FhuA, Llp, or FhuA–Llp were extracted with either 1.5% lauryldimethylamine oxide (LDAO) or 2.5% Elugent (typical volume ∼60 mL for membranes from 2 L of culture) by douncing, followed by stirring for 2 h at 4 °C or, in some cases, overnight. No difference was observed in extraction efficiency or downstream purification, and no protease inhibitors were added. Following extraction, the suspension was centrifuged at 42,000 rpm for 30 min and the clarified extract was subjected to IMAC using Ni-charged chelating sepharose (Cytiva) equilibrated in TSB buffer plus 0.15% LDAO. Following loading, the column was washed with ∼20 column volumes of buffer with 30 mm of imidazole and eluted with approximately three column volumes of buffer with 200 mM imidazole. IMAC elutions were immediately concentrated by centrifugal filtration (Amicon Ultra-15; molecular weight cutoff [MWCO], 50 kDa for FhuA and 30 kDa for Llp) and subjected to SEC on Superdex-200 16/10 in (typically) 10 mM Hepes, 100 mM NaCl, 0.05% LDAO, pH 7.5, and appropriate peak fractions were collected. In the case of FhuA and Llp co-overexpression, preparations from cells grown at 37 °C showed, when analyzed via SEC, in addition to FhuA–Llp a peak for free Llp, indicating that Llp is present in excess to FhuA in the *E. coli* OM. For FhuA–Llp crystallization, detergent exchange was performed by a second SEC column in which the LDAO was replaced by either 0.4% C_8_E_4_ or 0.25% decyldimethylamine oxide (DDAO). Protein was concentrated to 10 to 15 mg/mL, flash frozen in liquid nitrogen, and stored at −80 °C. The FhuA–Llp preparation that gave the diffracting crystals was treated with TEV protease following SEC, using TEV buffer (50 mM Tris, 0.5 mM EDTA, 0.2 mM tris(2-carboxyethyl)phosphine, pH 8) containing 0.05% dodecylmaltoside. A ratio of TEV to FhuA–LLp of ∼10 (wt/wt) was used and the incubation was done at 4 °C for 16 h. Following cleavage, TEV was separated from FhuA–Llp via SEC in 0.4% C_8_E_4_, and protein was concentrated to 10 to 15 mg/mL, aliquoted, flash frozen in liquid nitrogen, and stored at −80 °C.

Following ultracentrifugation, the supernatant of pb5-expressing cells was loaded on IMAC and processed as above in the absence of any detergent. However, analysis on SEC (10 mM Hepes, 100 mM NaCl, pH 7.5) showed that pb5 eluted as a broad peak. We subsequently observed that adding detergent to the pb5 sample improved the behavior on SEC. We therefore added 0.1% LDAO to the supernatant followed by prepurified FhuA, ensuring that pb5 was in at least twofold excess over FhuA. Following a short (15 min) incubation, the supernatant was loaded on IMAC and processed as for free FhuA. The IMAC elution was concentrated (50-kDa MWCO cutoff) and loaded on a Superdex-200 16/60 column equilibrated in 0.05% LDAO as described above. For crystallization attempts and cryo-EM data collection, detergent exchange to 0.4% C_8_E_4_ or 0.12% decylmaltoside, respectively, was done via preparative SEC. Peaks corresponding to FhuA–pb5 and free pb5 were collected, concentrated to ∼8 to 10 mg/mL and 0.5 mg/mL, respectively, and flash frozen in liquid nitrogen. To improve the stability of free pb5, ∼10% glycerol was added prior to flash freezing.

For in vitro interaction studies, 1 to 1.5 nmol of each protein was mixed and incubated at room temperature for ∼15 min and run on a Superdex-200 Increase 10/300 GL equilibrated in 10 mM Hepes/100 mM NaCl, 0.05% LDAO, pH 7.5 (injection volume ∼0.4 mL, flow rate 0.5 mL/min).

### Cryo-EM Data Acquisition for FhuA–pb5 and Data Processing.

Purified FhuA–pb5 complex in DM (3.5 μL) at 8 mg/mL was applied to glow-discharged Quantifoil 1.2/1.3 300-mesh holey carbon grids. The grids were immediately blotted and plunge-frozen in liquid ethane using a Vitrobot Mark IV (ThermoFisher Scientific) device operating at 4 °C and ∼100% humidity. Data were collected on an FEI Titan Krios microscope operating at 300 kV using a Falcon 4 direct electron detector with a Selectris imaging filter (10eV slit width) (ThermoFisher Scientific) at the Astbury Biostructure Laboratory (*SI Appendix*, Table S1). A total of 8,387 movies were recorded in counting mode at ×165,000 magnification, corresponding to a pixel size of 0.71 Å.

All image processing was done in cryoSPARC, version 3.3.2 (44, 45). Movies were motion corrected using patch motion correction, and contrast transfer function (CTF) parameters were estimated using patch CTF estimation. A total of 6,566 micrographs remained after discarding average intensity, defocus, and ice-thickness outliers. Initially, ∼2,000 particles were picked manually, subjected to two-dimensional (2D) classification, and then the resulting 2D classes were used for template-based picking. A total of 2,054,732 particles were extracted in 360 pixel boxes. The 2D classification was used to discard bad particles, followed by generation of an ab initio three-dimensional model using a stochastic gradient descent approach with two classes. Particles from the class with clear extra-micellar density were subjected to nonuniform refinement, resulting in a 3.57-Å reconstruction from 74,313 particles. The particles were re-extracted with a box size of 440 pixels, and the final set of 71,476 particles was used in nonuniform refinement with CTF parameter (i.e., beam tilt and trefoil) and per-particle defocus estimation. The final reconstruction had a global resolution of 3.1 Å, with the protein core regions reaching 2.7 Å as assessed by local resolution estimation (*SI Appendix*, Fig. S2). The FhuA crystal structure and the AF2 model of pb5 were rigid-body fit in the map via Phenix DockinMap (46), and the model was built via several cycles of manual building in *Coot* (47) and real-space refinement within Phenix. The final model refinement statistics are listed in *SI Appendix*, Table S1. Inspection of EM density maps was performed using ChimeraX (48) and *Coot*. Figures of maps were generated using ChimeraX and figures of models were made with Pymol.

### Crystallization and Structure Determination of FhuA–Llp.

Preparations of FhuA–Llp purified in either DDAO or C_8_E_4_ were subjected to initial crystallization screening via sitting drop vapor diffusion, by mixing 200 nL of protein with 200 or 150 nL of well solution via a Mosquito crystallization robot (TTP Labtech) at 20 °C. Several commercial screens were typically used (e.g., MemGold, MemGold2, MemTrans, and MemChannel; Molecular Dimensions). Several hits were obtained in C_8_E_4_, but only one of these (16% PEG 4K, 0.4 M (NH_4_)_2_SO_4_, 0.1 M NaAc, pH 4.5) diffracted occasionally to beyond 5Å resolution. The size of the initial crystals was increased manually via hanging drop vapor diffusion with larger drops (typically 1 to 1.5 μL), and further fine screening around the initial hit condition led to the collection of a best dataset with moderately anisotropic diffraction to ∼3.4Å resolution. MR via Phaser (49) within Phenix (46) using FhuA as a search model (PDB 1BY3) gave a definite solution for FhuA and density on the periplasmic side of the plug, but this was of insufficient quality for model building. Addition of an AF2-predicted model for Llp provided a solution that allowed building of the complete model for Llp via several cycles of model building in *Coot* and refinement via Phenix. During refinement, automatically assigned Translation-Libration-Screw-rotation was used and X-ray/atomic displacement parameter weights were optimized to keep the refinement stable, resulting in tight RMSD values for the bond lengths and angles. Moreover, using data processed via AUTOSOL/STARANISO (50, 51) provided the best refinement results. Data collection and refinement statistics are summarized in *SI Appendix*, Table S2. Interaction surfaces were analyzed via the PISA webserver at https://www.ebi.ac.uk/msd-srv/prot_int/pistart.html.

### Molecular Dynamics Simulations.

Classical atomistic MD simulations were initiated from the resolved X-ray structure of the FhuA–Llp complex. The system setup was prepared using the CHARMM-GUI webserver (52). Lipid tails were added to the N-terminal cysteine residue of Llp, and the protein complex was embedded in an OM model from *E. coli* containing Ra-LPS without O antigen in the outer leaflet and 1-palmitoyl 2-cis-vaccenic phosphatidylethanolamine, 1-palmitoyl 2-cis-vaccenic phosphatidylglycerol, and cardiolipin (1-palmitoyl 2-cis-vaccenic 3-palmitoyl 4-cis-vaccenic diphosphatidylglycerol) in the inner leaflet in a molar ratio of 90:5:5. The system was solvated with water containing 200 mM KCl ions, and negatively charged chemical groups of LPS were neutralized with calcium ions. Three MD simulations were performed starting from this system setup. Energy minimization was conducted for 5,000 steps, using the steepest descent algorithm. Six short, consecutive MD simulations of 20 ns in total were performed following the CHARMM-GUI protocol employing an integration timestep of 1 to 2 fs, the Berendsen thermostat and barostat at a temperature of 313 K, and a pressure of 1 bar (53), and decreasing position restraints of amino acids and lipids. MD simulations of the three equilibrated systems were performed for 2 μs each using an integration timestep of 2 fs, the Verlet cutoff scheme, and a cutoff distance for van der Waals and electrostatic interactions of 1.2 nm. The Nosé–Hoover thermostat was employed at a temperature of 313 K (54, 55), and semi-isotropic pressure coupling was achieved using the Parrinello–Rahman barostat at a pressure of 1 bar (56). Covalent bonds involving hydrogen atoms were constrained using the LINCS algorithm (57). Electrostatic interactions were evaluated using the particle-mesh Ewald method (58). MD simulations were performed using the GROMACS software package (59) and the CHARMM36m force field (60, 61) and were visualized and analyzed using the VMD software (62). Simulations starting from the AF2-predicted complex were run following the exact same protocol but without the lipid anchor on Llp Cys1 and for 500-ns durations.

## Supplementary Material

Supplementary File

Supplementary File

Supplementary File

## Data Availability

Original data created for the study are available in a persistent repository upon publication. Coordinates and structure factors that underlie the findings of this work have been deposited in the Protein Data Bank with accession codes 8A60 (FhuA–Llp) (36) and 8A8C (FhuA-pb5) (29). Electron density maps have been deposited in the Electron Microscopy Data Bank with accession code 8A8C and EMD-15229 (30).
