# Supplementary material for: Structural basis for host recognition and superinfection exclusion by bacteriophage T5
Source: Proc Natl Acad Sci U S A. 2022 Oct 10;119(42):e2211672119. doi: 10.1073/pnas.2211672119 (PMC9586334; doi:10.1073/pnas.2211672119)
Supplement: Supplementary File [file pnas.2211672119.sapp.pdf]

PNAS

Supporting Information for

Structural basis for host recognition and superinfection exclusion by bacteriophage T5

Bert van den Berg, Augustinas Silale, Arnaud Baslé, Astrid F Brandner, Sophie L. Mader, Syma Khalid

Corresponding author: Bert van den Berg
Email: bert.van-den-berg@ncl.ac.uk

This PDF file includes:

Figures S1 to S11
Tables S1 to S2
Legends for Movies S1 to S2

Other supporting materials for this manuscript include the following:

Movies S1 to S2

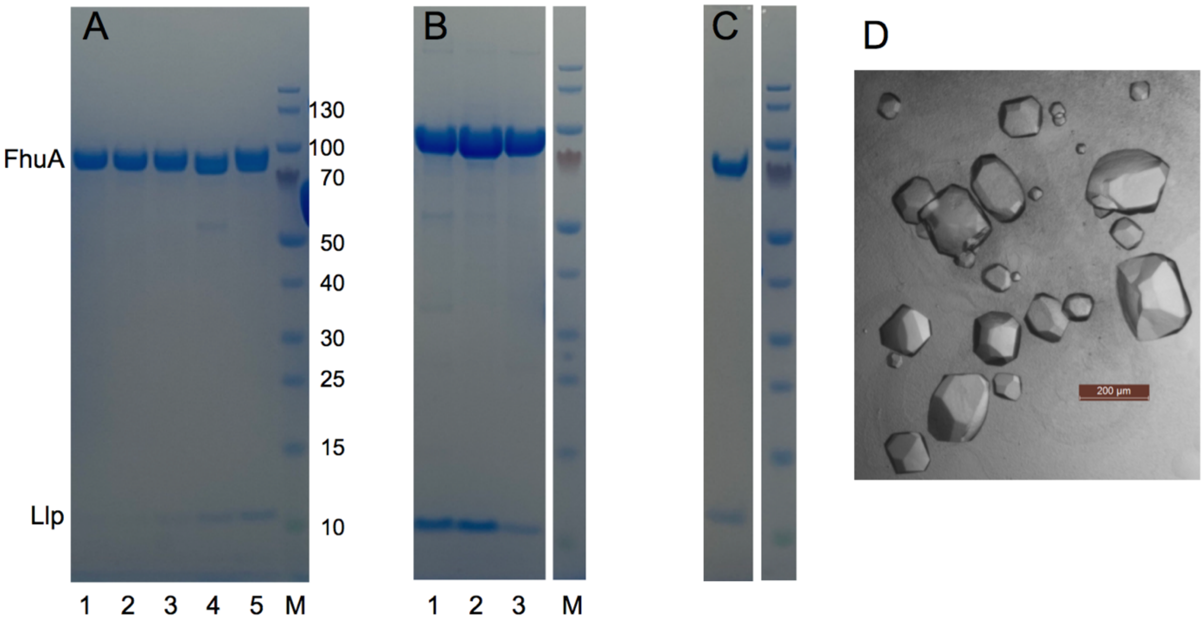

Fig. S1. Various properties of the FhuA-Llp complex explored via SDS-PAGE. (A) Complex formation *in vitro* is extremely slow. Purified Llp was incubated at 2-fold molar excess to FhuA and samples were analysed via SEC (Superdex-200 Increase 10/300 GL) at various time points. Lane 1, 30 min; lane 2, 60 min; lane 3, 24 hrs; lane 4, 48 hrs; lane 5, *in vivo*-generated FhuA-Llp complex purified in LDAO; M, molecular weight marker. In all cases, a sample corresponding to the FhuA peak was analysed. Due to the small size of Llp (~7 kDa), FhuA and FhuA-Llp elute at the same volume. (B) Variability of the FhuA:Llp ratio for different preparations, which is most likely due to different relative co-expression levels of FhuA and Llp. Lane 1, stoichiometric amounts of separately-expressed FhuA and Llp, based on individual OD_{280}; lane 2, co-expressed FhuA-Llp preparation x; lane 3, co-expressed FhuA-Llp preparation y. (C) Dissolved FhuA-Llp crystals show the presence of both components. (D) Diffracting crystals of FhuA-Llp obtained in MemGold condition F12.

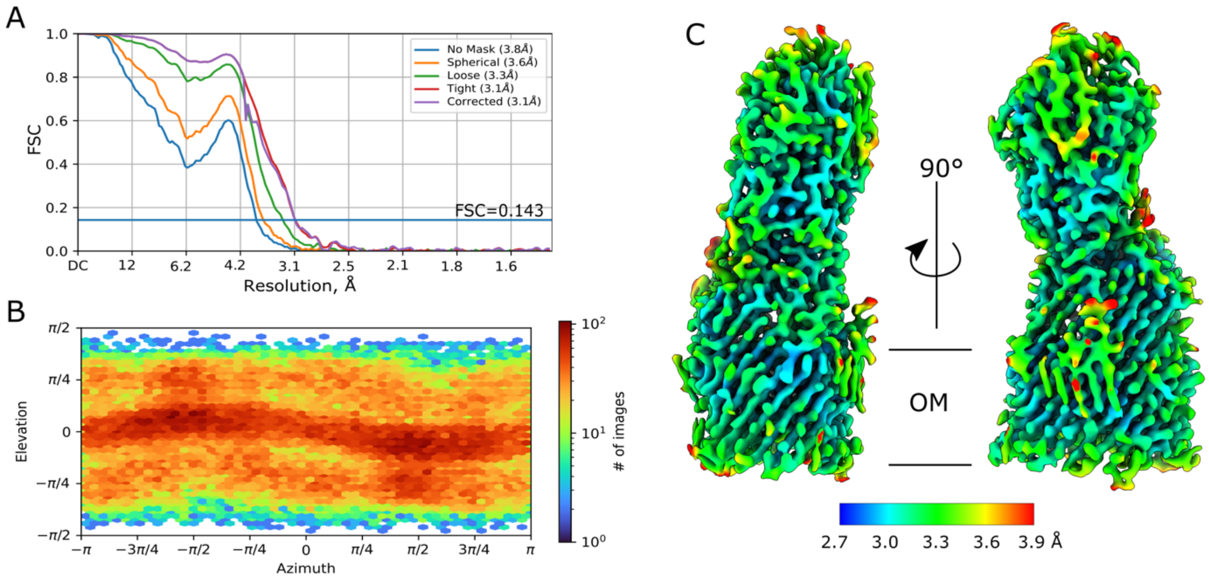

Fig. S2. Structure determination of FhuA-pb5. Gold-standard FSC curves (A) and viewing distribution plot (B). (C) Local resolution map of FhuA-pb5.

A

Rank	Chain	Z	rmsd	lali	nres	%ID	Description
1	2gsy-E	5.3	3.5	99	433	10	Polyprotein
2	2vme-A	4.5	3.7	88	256	7	Discoidin-2
3	2frg-P	4.1	3.2	72	106	7	Trem-like Transcript-1
4	4qxl-A	3.9	3.3	82	115	4	Flagellar protein FlhE
5	2wq4-A	3.9	2.7	78	134	10	Lectin
6	4y9v-A	3.8	9.4	103	603	6	Particle-associated Lyase
7	2cgz-A	3.7	3.4	78	101	5	Agglutinin
8	3o61-A	3.6	4.0	45	187	2	GDP-Mannose pyrophosphatase NudK
9	1qex-A	3.6	7.6	86	288	10	Bacteriophage T4 GP9
10	3wmp-A	3.6	3.6	77	94	5	Galactose-binding Lectin

B

Rank	Chain	Z	rmsd	lali	nres	%ID	Description
1	3av4-A	3.9	2.8	45	1140	18	DNA (Cytosine-5)-Methyltransferase 1
2	6vil-C	3.8	3.1	45	153	11	BAH and coiled-coil containing protein 1
3	6w8v-b	3.8	3.0	45	826	18	DNA (Cytosine-5)-Methyltransferase 1
4	4yoc-A	3.8	2.6	45	882	16	DNA (Cytosine-5)-Methyltransferase 1
5	6x9i-A	3.8	2.8	45	816	16	DNA (Cytosine-5)-Methyltransferase 1
6	4dov-A	3.7	2.6	45	157	9	Origin Recognition Complex Subunit 1
7	2o2w-A	3.7	2.4	45	67	9	Ponsin
8	2o9v-A	3.7	2.7	45	67	9	Ponsin
9	5wy1-A	3.7	2.8	45	1140	18	DNA (Cytosine-5)-Methyltransferase 1
10	6w8w-B	3.7	3.0	45	827	18	DNA (Cytosine-5)-Methyltransferase 1

Fig. S3. DALI analyses show low structural similarity of both pb5 and Llp to other proteins. Top 10 hits are shown for pb5 (A) and Llp (B). Coordinates were submitted to the webserver at http://ekhidna2.biocenter.helsinki.fi/dali/.

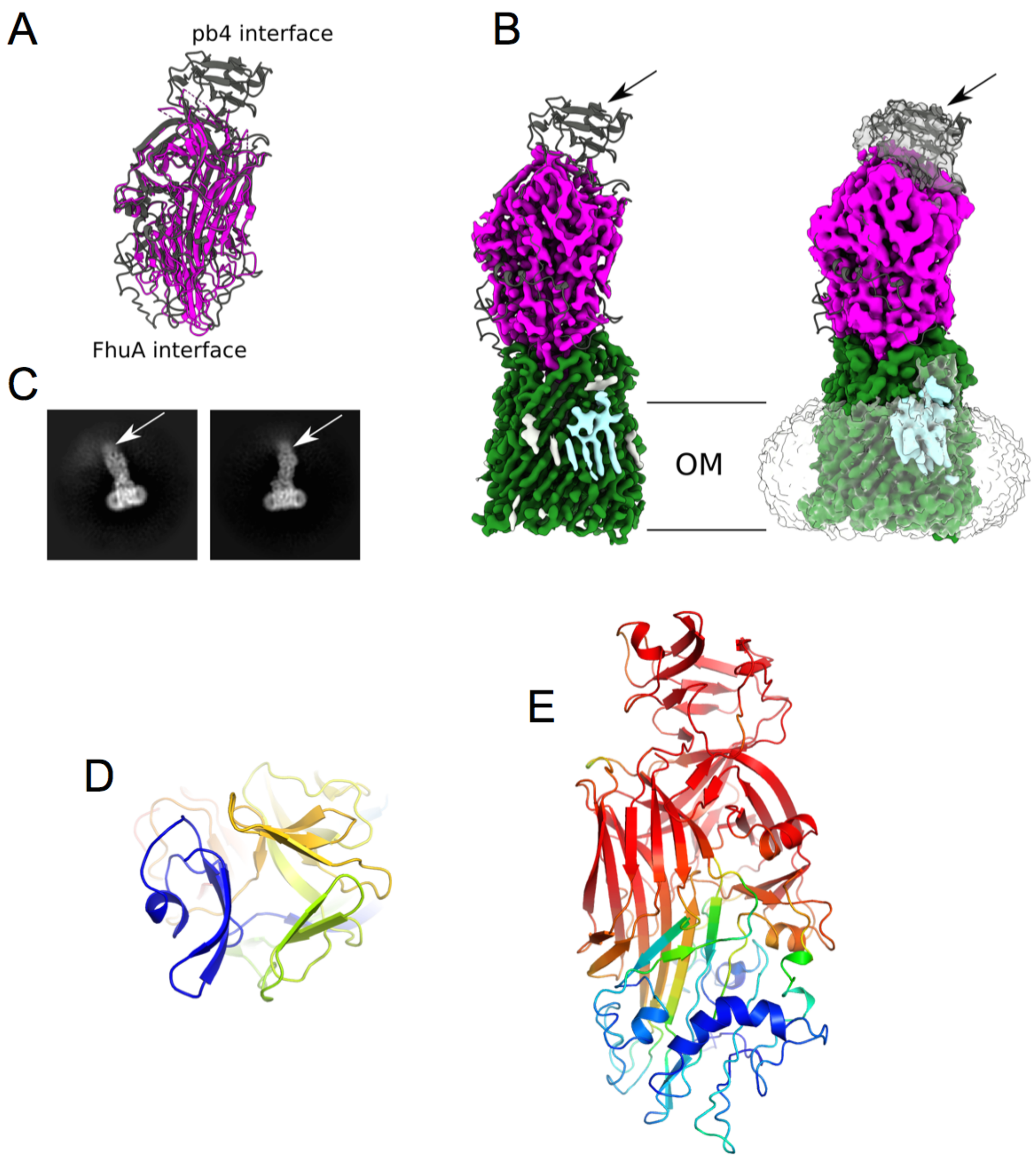

Fig. S4. The pb4-interacting domain of pb5 is disordered in the FhuA-pb5 cryo-EM structure. (A) Overlay of the AlphaFold2 prediction of pb5 (dark gray) with the experimental structure (magenta). (B) Low threshold cryo-EM map on the right showing the pb4-interacting domain (arrow). Micelle density is transparent. (C) Representative 2D classes for FhuA-pb5, showing fuzzy density for the pb4-interacting domain of pb5 (arrow). (D) Top-down view of the pb4-interacting domain from the AlphaFold2 prediction, showing the pseudo 3-fold symmetry. (E) Pb5 AlphaFold2 prediction coloured based on pLDDT confidence values (red, high; blue, low; minimum and maximum values are 24 and 97 respectively).

A

Structure 1			Structure 2			Interface	Δ^iG	N_{HB}	N_{SB}	N_{SB}	CSS
$^iN_{at}$	$^iN_{res}$	Surface Å^2	$^iN_{at}$	$^iN_{res}$	Surface Å^2	Area, Å^2	kcal/mole				
217	63	15867	230	62	25496	2116.8	-21.9	27	0	0	1.000

Hydrogen bonds

Structure 1	Dist. (Å)	Structure 2	Structure 1	Dist. (Å)	Structure 2
Arg 167 (NH2)	2.37	Tyr 315 (OH)	Gly 589 (N)	3.01	Ala 465 (O)
Arg 168 (NE)	3.05	Phe 557 (O)	Ser 590 (OG)	3.86	Thr 467 (OG1)
Arg 168 (NE)	3.58	Phe 558 (O)	Phe 170 (O)	2.59	Tyr 315 (OH)
Arg 168 (NH2)	2.58	Ser 559 (OG)	Gly 191 (O)	3.02	Lys 327 (N)
Phe170 (N)	2.94	Tyr 315 (OH)	Tyr 192 (O)	3.70	Lys 327 (NZ)
Ser 172 (OG)	3.65	Asp 394 (OD2)	Ala 171 (O)	2.46	Lys 344 (NZ)
Ser 173 (N)	3.38	Asp 394 (OD2)	Phe 170 (O)	3.12	Lys 344 (NZ)
Ser 190 (OG)	3.15	Asp 395 (O)	Ala 171 (O)	3.60	Asp 394 (N)
Tyr 192 (OH)	3.41	Asp 394 (OD2)	Ser 190 (OG)	2.67	Asn 419 (ND2)
Ser 265 (OG)	3.41	Pro 321 (O)	Gln 115 (OE1)	3.13	Tyr 423 (OH)
Tyr 268 (OH)	3.40	Tyr 325 (OH)	Asn 578 (OD1)	2.63	Asn 462 (ND2)
Ser 285 (OG)	3.11	Glu 554 (OE1)	Thr 240 (OG1)	3.07	Gly 555 (N)
Tyr 577 (OH)	3.87	Val 506 (O)	Gln 119 (OE1)	3.28	Phe 557 (N)
			Glu 264 (OE1)	3.58	Tyr 696 (OH)

B

Structure 1			Structure 2			Interface	Δ^iG	N_{HB}	N_{SB}	N_{SB}	CSS
$^iN_{at}$	$^iN_{res}$	Surface Å^2	$^iN_{at}$	$^iN_{res}$	Surface Å^2	Area, Å^2	kcal/mole				
153	37	4418	178	47	27761	1641.8	-15.0	16	4	0	1.000

Hydrogen bonds

Structure 1	Dist. (Å)	Structure 2	Structure 1	Dist. (Å)	Structure 2
Tyr 15 (OH)	3.30	Gln 44 (OE1)	Ser 44 (OG)	2.94	Glu 57 (OE1)
Gln 17 (NE2)	3.61	Gln 44 (OE1)	Trp 46 (NE1)	3.64	Asn 583 (OD1)
His 19 (ND1)	3.36	Glu 289 (OE2)	Tyr 15 (OH)	3.80	Gln 44 (N)
His 19 (NE2)	3.09	Asp 287 (OD2)	Gln 17 (OE1)	2.97	Gln 44 (NE2)
Lys 23 (N)	3.20	Pro 42 (O)	Asp 18 (OD1)	2.59	Arg 297 (NH2)
Asn 25 (ND2)	3.50	Glu 530 (O)	Asp 18 (OD1)	2.65	Gln 362 (NE2)
Tyr 42 (OH)	2.33	Glu 56 (OE1)	Ile 39 (O)	2.94	Ser 49 (OG)
Ser 44 (OG)	3.09	Thr 54 (OG1)	Tyr 42 (O)	2.36	Arg 128 (NH1)

Salt bridges

Structure 1	Dist. (Å)	Structure 2	Structure 1	Dist. (Å)	Structure 2
His 19 (ND1)	3.36	Glu 289 (OE2)	Asp 18 (OD1)	2.59	Arg 297 (NH2)
His 19 (NE2)	3.09	Asp 287 (OD2)	Asp 18 (OD2)	3.43	Arg 297 (NH2)

Fig. S5. PISA analyses for FhuA-pb5 (A) and FhuA-Llp (B). The upper panels show an overview of the relevant interfaces. CSS stands for complexation significance score (maximum value 1.000). In both cases, FhuA is Structure 2.

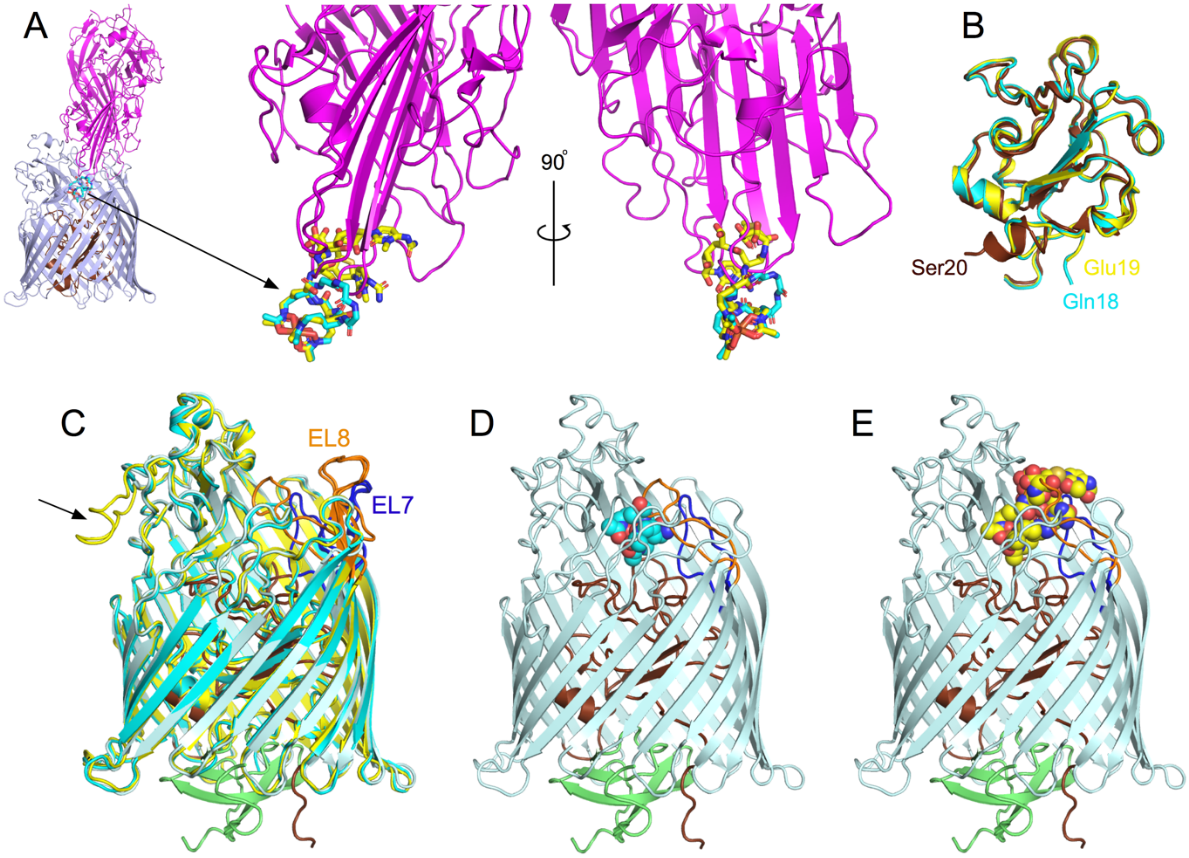

Fig. S6. Pb5 and Llp binding to FhuA abolishes binding (and transport) of the ligands ferrichrome and albomycin. (A) Cartoon model for FhuA-bound pb5 in two orientations, showing the overlap of pb5 loops with ferrichrome (cyan sticks) and albomycin (yellow sticks) bound to FhuA (PDB IDs 1BY5 and 1QKX respectively). Left panel shows an overview for orientation. (B) Superpositions of the FhuA plug domains for FhuA-pb5 (brown), FhuA-ferrichrome (cyan), and FhuA-albomycin (yellow). The N-terminal residues visible in the structures are labeled. (C) Superposition of FhuA-Llp (light blue, with Llp in lime green) with FhuA-ferrichrome (cyan) and FhuA-albomycin (yellow). For clarity, the ligands are not shown. Loops EL7 are EL8 are coloured blue and orange, respectively. The arrow indicates the location of the internal His tag present in 1QKX. (D,E) FhuA-Llp with superposed ferrichrome (D) or albomycin (E) shown as space-filling models. In both cases, the inward-folded FhuA loops EL7 and EL8 clash with the ligands.

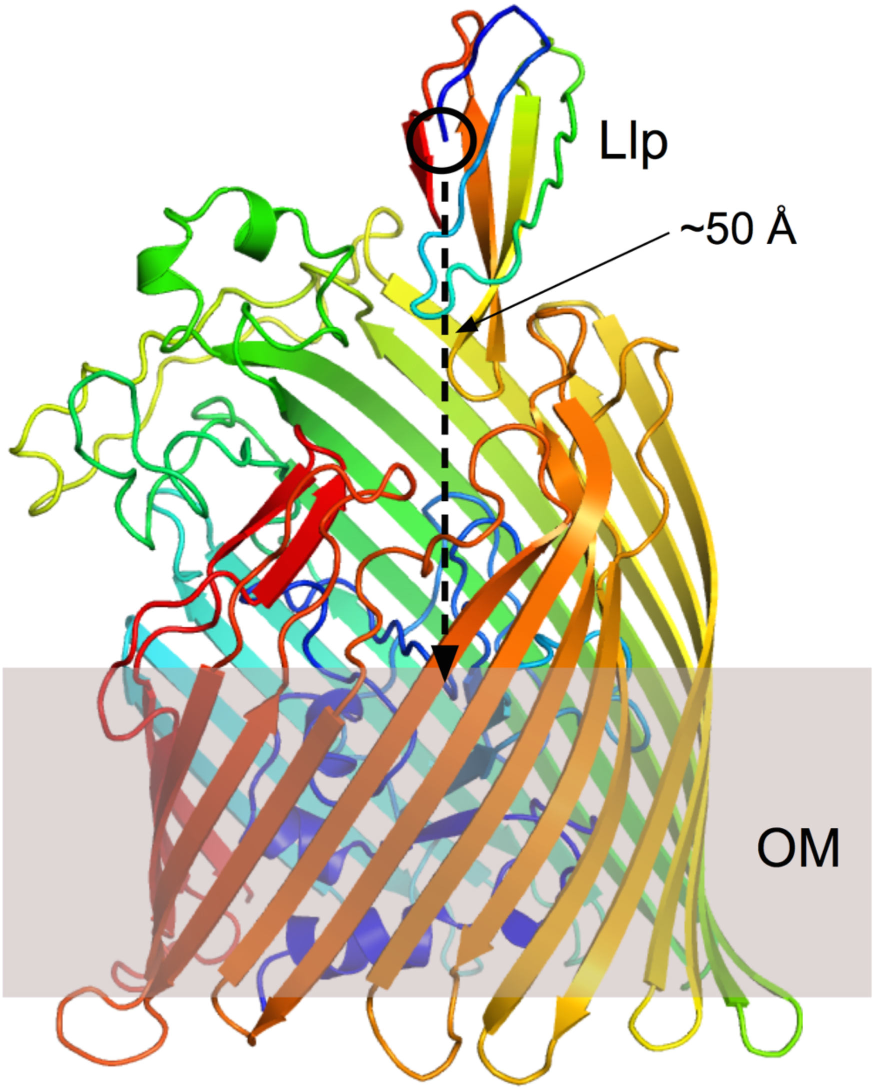

Fig. S7. AlphaFold2 prediction of the FhuA-Llp complex. Both proteins are shown in rainbow representation (N-terminus, blue). The location of the hydrophobic core of the OM is indicated. The location of Cys1 in Llp is indicated with a black circle, showing that it is at least 50 Å away from its expected location within the OM (dashed arrow).

A

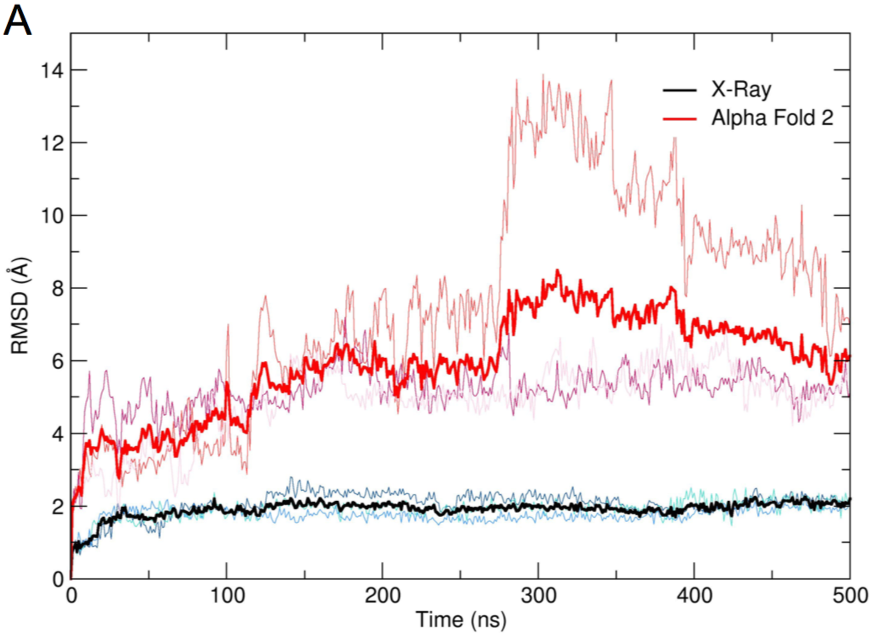

B

	Replica 1	Replica 2	Replica 3
% CC XRAY	79.02 (6.46)	76.63 (5.25)	74.29 (5.87)
% CC AF	70.49 (15.65)	57.03 (7.30)	69.53 (5.45)
% Acc XRAY	92.57 (3.41)	84.88 (4.52)	85.27 (3.53)
% Acc AF	54.68 (16.30)	63.97 (7.34)	58.5 (7.57)

Fig. S8. Computational analyses of FhuA-Llp complexes. (A) Llp Cα r.m.s.d values relative to the starting structures for three unbiased 500 ns molecular dynamics simulations. Three independent replicas are shown for the AF prediction (red) and for the X-ray structure (blue). The average curve is shown in bold (red, AF; black, X-ray). (B) Average contact conservation (CC) and accuracy (Acc) values of the simulations. Contact conservation is defined as the number of contacts at time t that was also present in the initial reference structure (true positives; TP), divided by the total number of contacts in the initial structure. A contact is defined as an atom-center to atom-center distance smaller than 4 Å, evaluated every 2 ns. Accuracy is defined as TP/(TP+FP), with FP being the number of new contacts appearing during the simulation (*i.e*. false positives FP). Values in parentheses are standard deviations. The analysis suggests that Llp is less stably bound in the AF-predicted complex compared to the crystal structure.

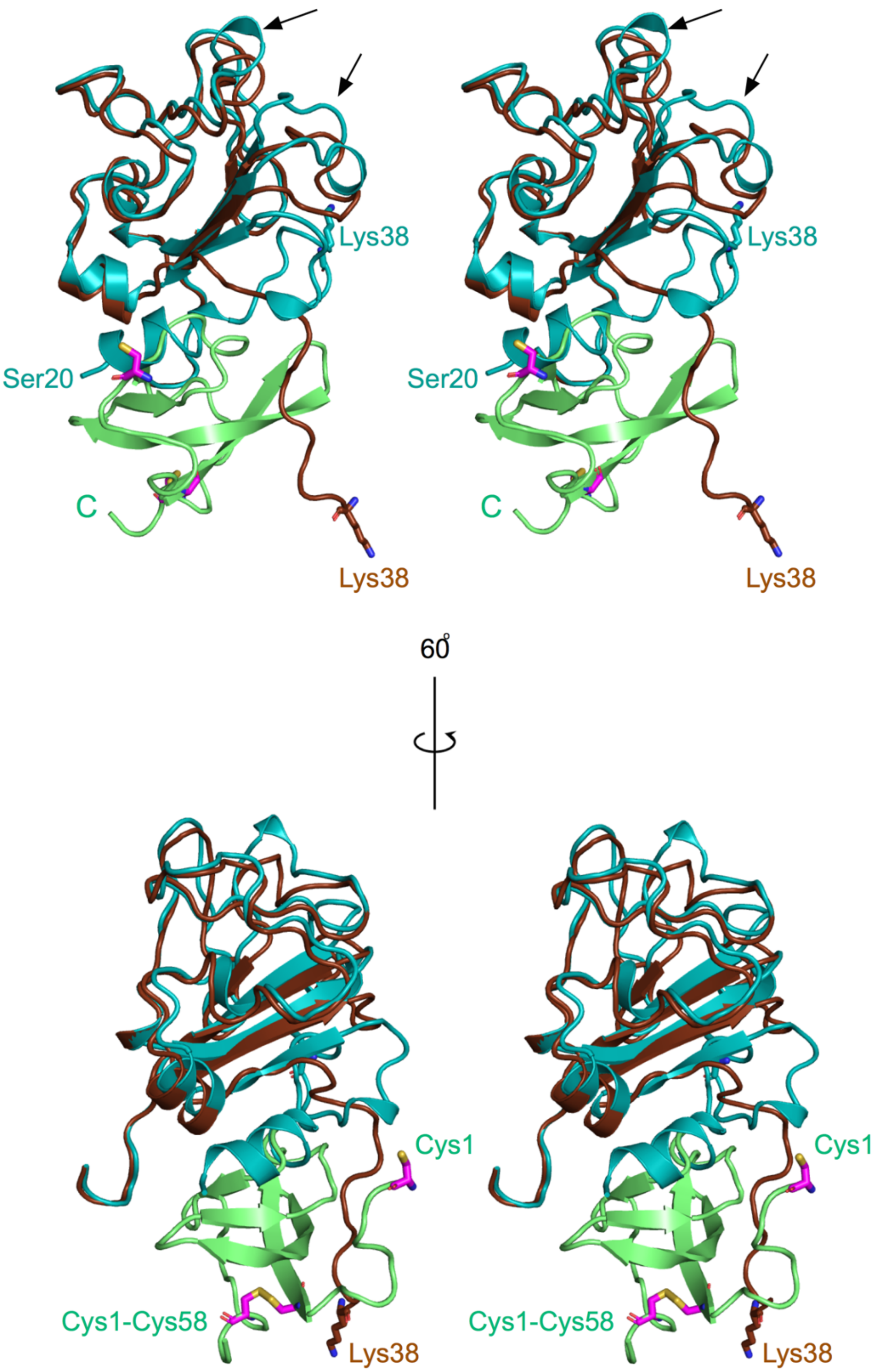

Fig. S9. Conformational differences in the plug of FhuA-Llp. Stereo superpositions of the free FhuA plug (teal) and the plug of FhuA-Llp (brown). Cysteine residues of Llp (lime green) are shown as magenta stick models. The visible N-termini are labelled and correspond to Ser20 in free FhuA and Lys38 in FhuA-Llp. The periplasmic space is at the bottom of the figure. The arrows in the top panel point to conformational changes in the extracellular face of the FhuA plug that likely have propagated from the Llp binding site.

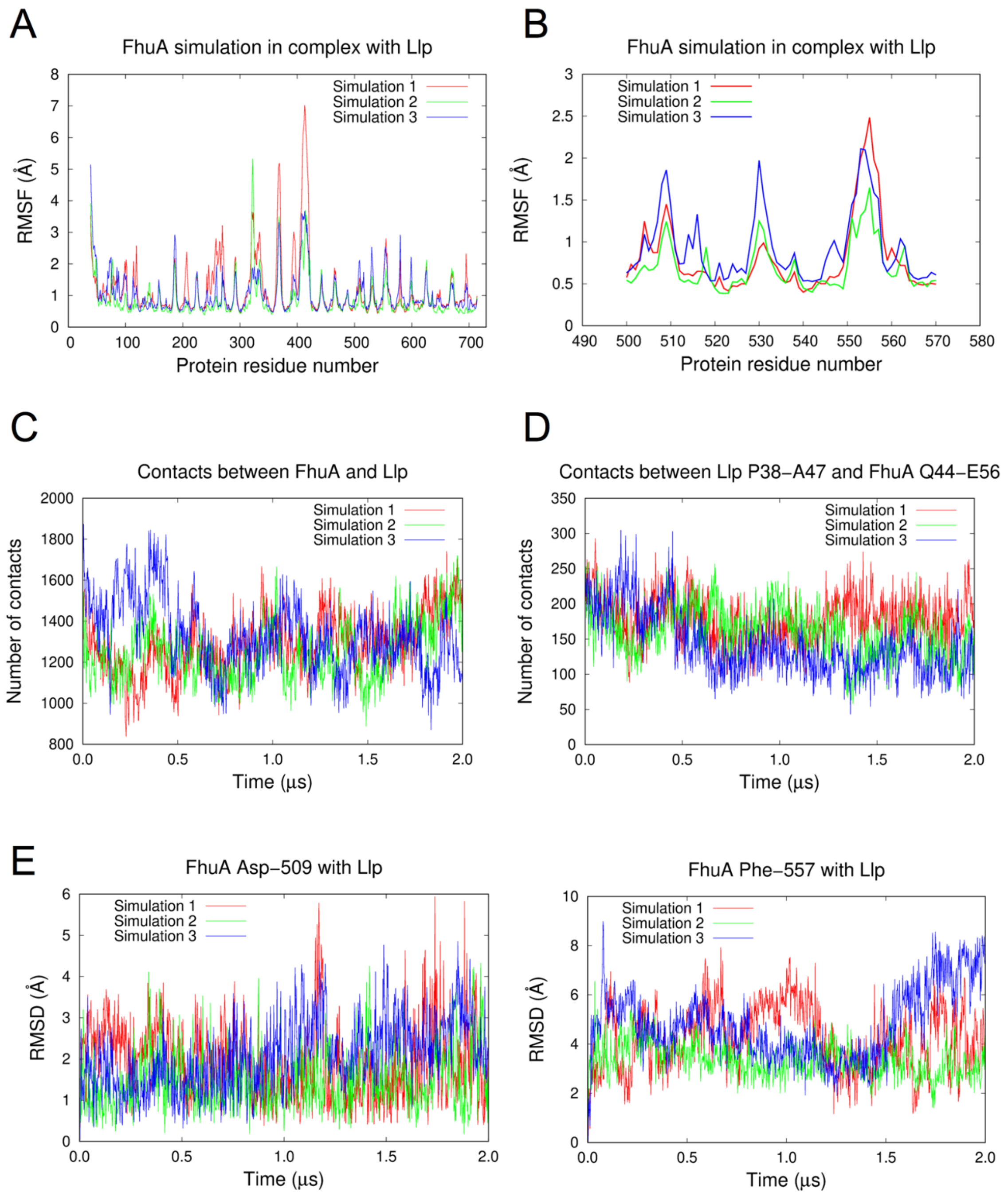

SI Fig. 10. The FhuA-Llp interaction is stable and the conformations of EL7 and EL8 are likely not caused by crystal contacts. MD simulations for the FhuA-LLp crystal structure showing Cα root-mean-square-fluctuations for FhuA (A) and the EL7-EL8 region (B). (C,D) Total number of contacts between FhuA and Llp (C) and between Llp Pro38-Ala47 and FhuA plug residues Gln44-Glu56 (D). A contact is defined as an atom-center to atom-center distance smaller than 4 Å, evaluated every 2 ns. (E,F) The positions of the tips of EL7 (Asp509; E) and EL8 (Phe557; F) are relatively stable during the 2 μs simulations compared to the starting structure.

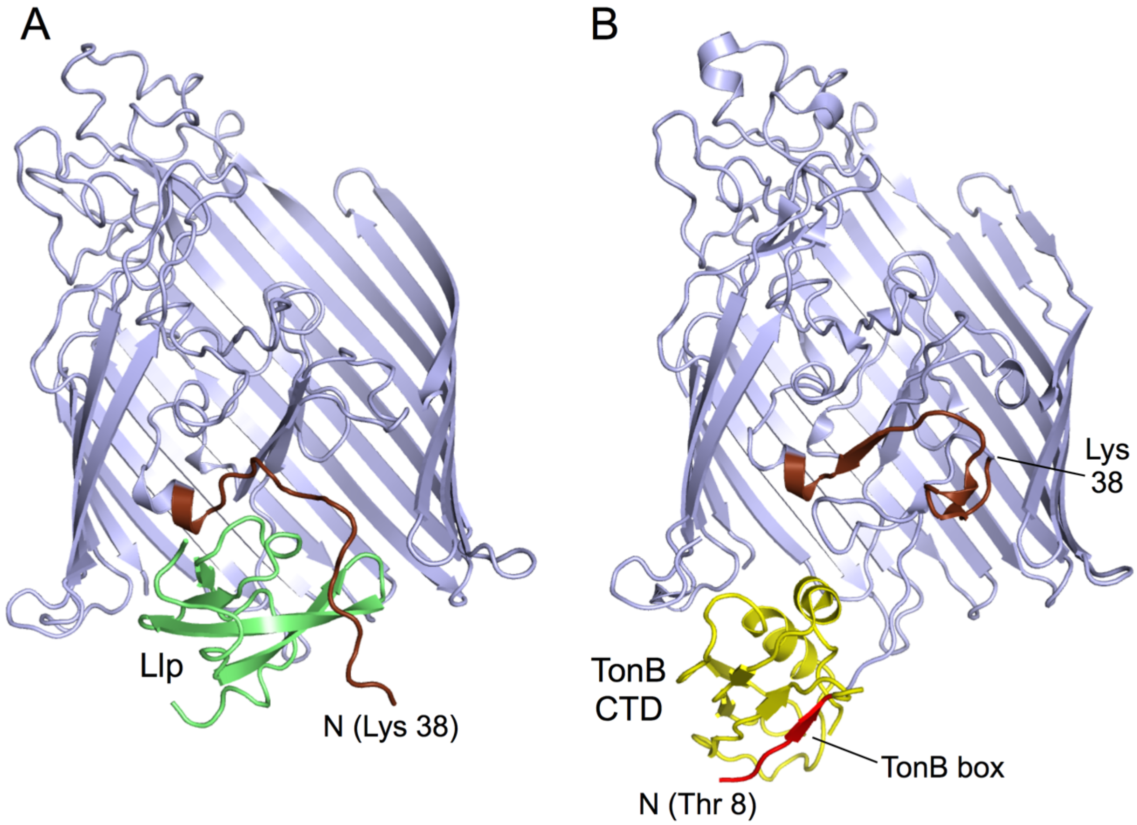

Fig. S11. The TonB box of FhuA in FhuA-Llp is not visible but likely accessible for interaction with TonB. Side-by-side comparison of FhuA-Llp (A) and the complex of FhuA with the C-terminal domain (CTD; yellow) of TonB (PDB ID 2GRX; ref. 1) (B). The FhuA plug segment Lys38-Glu57 is coloured brown. The TonB box of FhuA corresponds to the segment Asp7-Ala13 and is coloured red. For clarity, part of the FhuA barrel wall has been removed in both panels.

Table S1. Cryo-EM data acquisition, processing and FhuA-pb5 model parameters.

**Data collection**	
Electron microscope	Titan Krios
Voltage (kV)	300
Spherical aberration (μm)	2.7
Camera	Falcon 4
Energy filter	Selectris (10 eV slit)
Magnification	165,000
Pixel size (Å)	0.71
Total dose (e^-/ $Å^2$)	40.1
Dose rate (e^-/pixel/s)	7.37
Defocus minimum, maximum (μm)	-1.0, -2.0
Number of EPU frames	94
Number of movies collected	8,387
**Image Processing**	
Motion-corrected micrographs used	6,566
Initial number of particles	2,054,732
Final number of particles	71,476
Global resolution (FSC = 0.143)	3.1 Å
**Refinement**	
Model composition	
Non-hydrogen atoms	9550
Protein residues	1215
R.m.s. deviations	
Bond lengths (Å)	0.617
Bond angles (°)	0.006
Validation	
Molprobity score	2.2
Clash score	16.2
Rotamer outliers (%)	0.6
Ramachandran plot	
Favoured (%)	92.3
Outliers (%)	0.2
PDB	8A8C
EMDB	EMD-15229

Table S2 X-ray crystallographic data collection and refinement statistics for FhuA-LIp

**Data collection**#	
Wavelength (Å)	0.979
Space group	$P3_221$
Cell dimensions (Å)	
a, *b*, *c* (Å)	98.3, 98.3, 236.6
α, β, γ (°)	90, 90, 120
Resolution (Å)	85.1-3.37 (3.61-3.37)*
R_{merge}	0.28 (2.85)
R_{pim}	0.076 (0.80)
$I / \sigma I$	9.2 (1.7)
CC1/2	1.00 (0.39)
Completeness spherical (%)	78.1 (20.2) ##
Completeness ellipsoidal (%)	87.4 (48.0)
Redundancy	14.1 (13.0)
No. unique reflections	15120 (697)
**Refinement**	
Resolution (Å)	57.9-3.37
No. reflections	15002
R_{work} / R_{free} (%)	25.3/30.7
No. atoms	5804
B-factors (average)	117.3
R.m.s. deviations	
Bond lengths (Å)	0.002
Bond angles (°)	0.55
Clashscore	11.1
Rotamer outliers (%)	0.0
Ramachandran plot	
Favoured (%)	86.8
Outliers (%)	2.6
PDB ID	8A60

# One crystal was used for data collection.

* Values in parentheses are for highest-resolution shell.

Diffraction limits & principal axes of ellipsoid fitted to diffraction cut-off surface:

3.588	1.0000	0.0000	0.0000	0.894 _a_* - 0.447 _b_*
3.588	0.0000	1.0000	0.0000	_b_*
3.373	0.0000	0.0000	1.0000	_c_*

Criteria used in determination of diffraction limits: local(I/sigI) >= 1.20

Movie S1. Pb5 inserts four loops into the extracellular FhuA lumen. OM plane view with FhuA coloured light blue and pb5 gray. Loops EL7 and EL8 of FhuA are dark blue and orange, respectively. Loops L1-L4 of pb5 are coloured as in Fig. 2B. Residues Gln115/Phe 170 of pb5 and Phe115/Tyr116 of the FhuA plug are shown as stick models.

Movie S2. Conformational changes in FhuA as a result of Llp binding. Morphs between free FhuA (PDB ID 1BY3) and FhuA-Llp are shown from the OM plane and from the extracellular side. FhuA loops EL7 and EL8 are blue and orange, respectively, while Llp is lime green. Residues Lys38-Glu57 of the plug are coloured pink.

Supplementary reference

1. P.D. Pawelek et al., Structure of TonB in complex with FhuA, E. coli outer membrane receptor. *Science* **312**, 1399-402 (2006).
